# A Prospective Observational Study on the Efficacy and Safety of Infliximab-Biosimilar (CT-P13) in Patients With Takayasu Arteritis (TAKASIM)

**DOI:** 10.3389/fmed.2021.723506

**Published:** 2021-09-27

**Authors:** Corrado Campochiaro, Alessandro Tomelleri, Silvia Sartorelli, Camilla Sembenini, Maurizio Papa, Federico Fallanca, Maria Picchio, Giulio Cavalli, Francesco De Cobelli, Elena Baldissera, Lorenzo Dagna

**Affiliations:** ^1^Unit of Immunology, Rheumatology, Allergy and Rare Diseases, Istituto di Ricovero e Cura a Carattere Scientifico (IRCCS) San Raffaele Hospital, Milan, Italy; ^2^Vita-Salute San Raffaele University, Milan, Italy; ^3^Unit of Radiology, IRCCS San Raffaele Hospital, Milan, Italy; ^4^Unit of Nuclear Medicine, IRCCS San Raffaele Scientific Institute, Milan, Italy

**Keywords:** Takayasu arteritis, biosimilar drug, infliximab, therapy, anti-TNF, biologic drug, bDMARD

## Abstract

**Objectives:** Infliximab (IFX) is widely used in patients with refractory Takayasu arteritis (TAK). Recently, the IFX-biosimilar CT-P13 has been introduced for the treatment of inflammatory diseases. The aim of this study was to assess the efficacy and safety of CT-P13 in patients with refractory TAK.

**Methods:** In this prospective, open-label, single-center trial, TAK patients either already on treatment with IFX-originator (switch group) or never treated with IFX (naïve group) received CT-P13 for 52 weeks. The primary outcomes of the study were: (i) number of patients with active disease at month 6; (ii) incidence of treatment-emergent adverse events at month 12. Disease activity was assessed at month 6 and month 12 by clinical evaluation (ITAS-2020, ITAS-ESR, and ITAS-CRP scores) and imaging assessment [magnetic resonance angiography (MRA) and (18F)-FDG-PET].

**Results:** 23 patients were recruited (21 switch, 2 naïve). At baseline, 7 patients (32%) were classified as active. At month 6, one patient voluntarily dropped out and 7 patients were still active (30%), including one patient started on a different bDMARD at month 2 due to poor disease control. Mean daily dose of prednisone equivalent was significantly lower than baseline (4.2 ± 1.9 mg vs. 4.8 ± 2.1 mg, *p* = 0.009). At month 12, another patient was excluded because of pregnancy desire. Five patients were classified as active (24%), including two patients started on a different bDMARD at month 2 and month 6. Mean daily dose of prednisone equivalent was significantly lower than baseline (3.3 ± 2.6, *p* = 0.034). No patient experienced side effects during CT-P13 infusion. Overall, one patient experienced grade 1 adverse event and 9 patients experienced grade 2 adverse events. In no case hospitalization was required. CT-P13 retention rate was 90.9% at month 6 and 90.4% at month 12.

**Conclusion:** In this study, the use of IFX-biosimilar CT-P13 in patients with refractory TAK showed satisfying efficacy and safety profile.

## Introduction

Takayasu arteritis (TAK) is a systemic vasculitis affecting the aorta and its branches, with a clear female predominance (1, 2). Chronic vascular inflammation may lead to arterial stenosis, occlusion, dilatation, and aneurysm formation (3). Glucocorticoids represent the cornerstone and the first-line approach in TAK patients (4). However, upon steroid tapering, more than 50% of patients experience a relapse, requiring the addition of immunosuppressive steroid-sparing agents (5, 6). Among these, anti-tumor necrosis factor α (aTNF) agents and the anti-interleukin-6 receptor tocilizumab, as monotherapy or in combination with a conventional synthetic disease modifying anti-rheumatic drug (csDMARD), represent the therapies of choice and their use has been included in the European recommendations for the management of patients with large-vessel vasculitis (4). Infliximab (IFX) is one of the five aTNF agents available to date and its use in refractory TAK is supported by several retrospective and observational studies (7–9). Additionally, our group recently highlighted how, together with golimumab, IFX was the biologic DMARD (bDMARD) with the highest retention rate in a large cohort of 50 TAK patients (10).

“Biosimilar drugs” are monoclonal antibodies highly similar and clinically equivalent to already approved biological agents which have been progressively introduced in the drug market over the last decade, especially in Europe (11). In 2015, the IFX-biosimilar (IFX-B) CT-P13 was approved by the European Medicine Agencies (EMA) for the treatment of rheumatoid arthritis, ankylosing spondylitis, psoriatic arthritis and inflammatory bowel diseases (12). In addition, it has also been evaluated as an *off-label* treatment for rare diseases and in 2018 Park and colleagues published the first promising results of a single-arm study on CT-P13 in 11 TAK patients (13). However, the study included only naïve patients and, due to the small number of patients included, it could not support the use of CT-P13 as an effective steroid-sparing agent in TAK.

The aim of this prospective observational study is to investigate the use of CT-P13 in refractory TAK, including patients who never received and patients already on treatment with IFX-originator (IFX-O).

## Methods

### Study Protocol

This is a spontaneous, open-label, 52-week, single-center, prospective trial investigating the efficacy and safety of CT-P13 (Remsima®) in TAK patients refractory to glucocorticoids and conventional immunosuppressive treatments (registration number: NCT03192878). The primary outcomes of the study are: (i) number of patients with active disease at month 6 (efficacy); (ii) incidence of treatment-emergent adverse events at month 12 (safety and tolerability). Secondary outcomes include: (i) number of patients with active disease at month 12; (ii) impact of the treatment on patients' quality of life as assessed by the Health Assessment Questionnaire (HAQ) at 6 and 12 months.

The study was conducted in accordance with the Declaration of Helsinki and to the existing legislation in the field of Observational Studies. Study participation was subject to the understanding and signing of a specific written informed consent. Enrolled patients could withdraw consent at any moment. Patients agreed to the collection and use of their personal data.

### Study Population

Study population consisted of TAK patients already treated (*switch* patients) or never treated (*naïve* patients) with IFX-O. All patients already on IFX-O, those who needed to start IFX as first biologic therapy, and those who needed to start IFX as an alternative to another biologic drug were offered the opportunity to take part in this study. Inclusion criteria were: (i) age ≥ 18 years; (ii) negative pregnancy test; (iii) use of a reliable contraceptive method by all potentially fertile patients during the study and for the 6 months following the end of study; (iv) fulfillment of the 1990 American College of Rheumatology classification criteria (14); (v) multifocal aortic and/or arterial vascular inflammation, as disclosed by at least one imaging investigation [i.e., angiography, magnetic resonance angiography (MRA), vascular ultrasound, or ^18^F-fluorodeoxyglucose positron emission tomography (FDG-PET)]. *Naïve* patients were considered eligible if they showed at least one of the following features: inadequate response after 4 weeks of prednisone therapy at a dose of 1 mg/Kg daily; impossibility to reduce the dose of prednisone to 0.5 mg/Kg daily within 3 months; impossibility to reduce the dose of prednisone to <0.2 mg/Kg daily within 6 months; impossibility to reduce the dose of prednisone to 0.5 mg/Kg daily within 3 months or to <0.2 mg/kg daily within 6 months despite concomitant therapy with cyclophosphamide, azathioprine, methotrexate, cyclosporin A, mycophenolate mofetil, leflunomide, or rapamycin for at least 3 months. *Switch* patients were considered eligible if they were already on treatment with IFX-O.

Exclusion criteria were: (i) history of lymphoproliferative disease or solid neoplasm in the previous 5 years, with the exception of successfully treated and completely resolved squamous cell skin carcinoma; (ii) history of uncontrolled diabetes, unstable cardiac ischaemia, congestive heart failure (NYHA class III and IV), active intestinal inflammatory disease, active peptic ulcer, recent stroke (within 3 months) and any other pathological condition that could expose the subject to the risk of adverse events according to the treating physician; (iii) serological tests for hepatitis B or hepatitis C indicating an active infection; (iv) history of HIV infection; (v) severe infections requiring hospitalization or treatment with intravenous antibiotic within 30 days prior to enrolment, or infections requiring treatment with oral antibiotics within 14 days prior to enrolment; (vi) ongoing pregnancy or lactation; (vii) history of drug or alcohol abuse; (viii) previous diagnosis of a demyelinating disease of the central nervous system; (ix) history of active tuberculosis, histoplasmosis or listeriosis; (x) previous *M. tuberculosis* infection, as documented by chest X-ray and positive QuantiFERON-TB Gold test (in this case, enrolment was allowed only after consultation with an infectious disease specialist and introduction of a prophylactic therapy).

### Study Drug

In *naïve* patients, IFX-B CT-P13 was administered intravenously at a starting dose of 5 mg/kg at weeks 0, 2, and 6, and then every 6 weeks. In *switch* patients, CT-P13 was administered intravenously with the same dosage of the previous IFX-O therapy. CT-P13 dose was allowed to be increased by 1 mg/kg up to a maximum of 10 mg/kg as per clinical judgment. Concomitant use of systemic glucocorticoids and csDMARDs was allowed. Active patients already on the highest tolerated dose of IFX-B could be switched to a different bDMARD as escape therapy.

### Study Assessments

**Figure 1:**
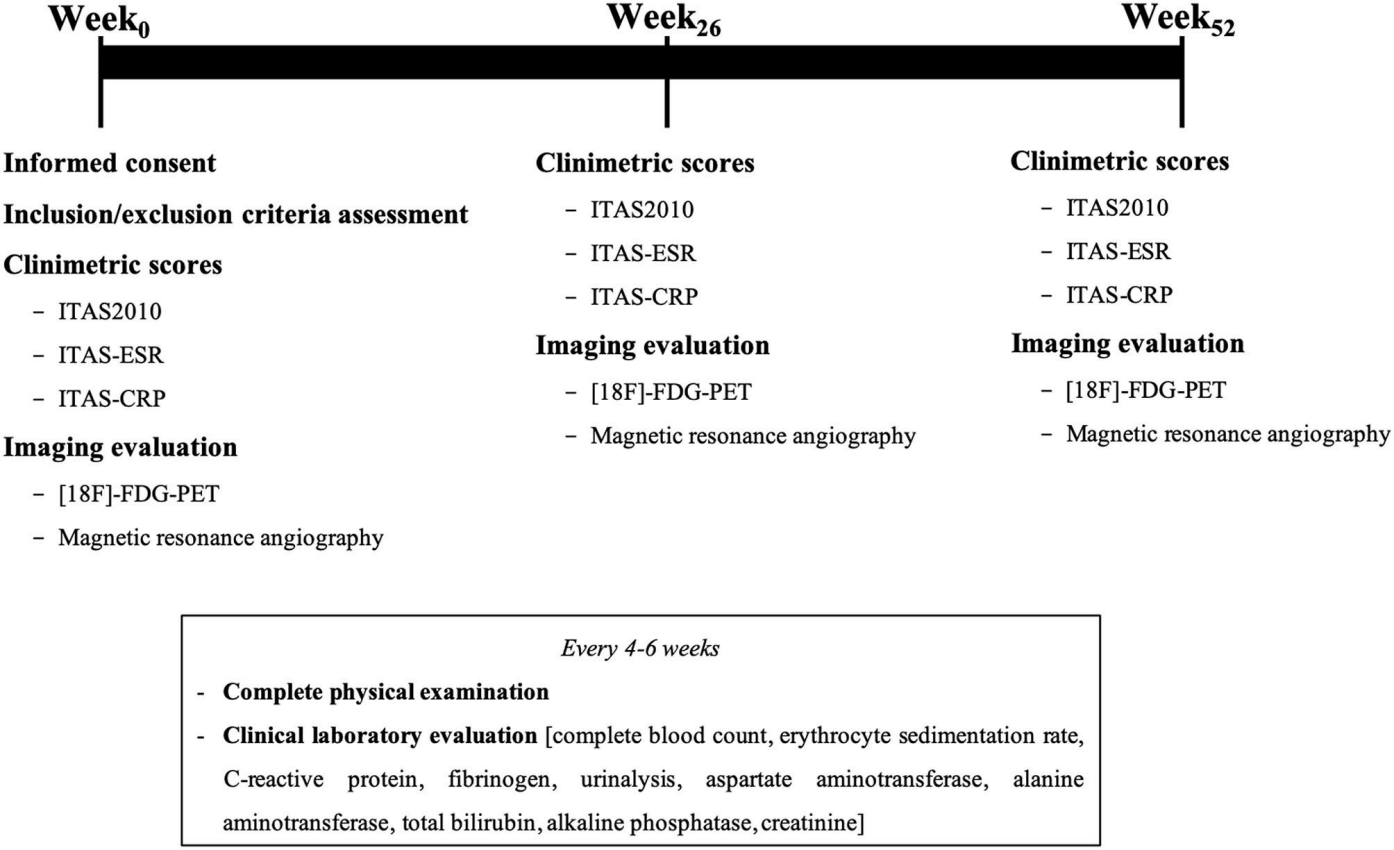
Timeline of study assessments.

All patients underwent clinical evaluation and laboratory tests at baseline and at the time of each infusion of the study drug, always before therapy administration. Clinical evaluation was always conducted by the same experienced physician (EB). It comprised collection of vital parameters, full physical examination and assessment of any sign or symptom suggestive of disease activity or adverse events. Laboratory tests always included complete blood count, dosage of acute-phase reactants [i.e., C-reactive protein (CRP), and erythrocyte sedimentation rate (ESR)], liver functions tests, serum creatinine, and urinalysis. All patients underwent both total-body MRA and total-body [^18^F]-FDG-PET at month 6 and month 12. See Figure 1.

TAK was defined active if a new vascular lesion or the worsening of at least one pre-existing lesion was detected by MRA and/or [^18^F]-FDG-PET. In addition, the following disease activity scores were used: Indian Takayasu Clinical Activity Score 2010 (ITAS2010), Indian Takayasu Clinical Activity Score – Erythrocyte Sedimentation Rate (ITAS-ESR) and Indian Takayasu Clinical Activity Score – C-Reactive Protein (ITAS-CRP). ITAS2010 ≥ 2, ITAS-ESR ≥ 3, or ITAS-CRP ≥ 3 were interpreted as expression of an active disease (15).

From the imaging perspective, disease was defined stable if both MRA and [^18^F]-FDG-PET disclosed no significant modifications of pre-existing vascular lesions and no appearance of new lesions; improved if they showed disappearance or reduction of extension (MRA) or of FDG uptake ([^18^F]-FDG-PET) of previously detected vascular lesions and absence of new vascular lesions.

Treatment-emergent adverse events were assessed according to *Common Terminology Criteria for Adverse Events* (CTCAE) v.4.

### Statistical Analysis

Data were analyzed with SPSS 24.0 (SPSS, Chicago, IL). Categorical variables were reported as numbers and percentages, whereas continuous variables were reported as the means ± standard deviation. Two-tailed Fisher's exact test and Wilcoxon-Signed Rank tests were used for statistical comparison. Survival analysis was performed with the Kaplan-Meier approach; comparison between survival curves was performed with the log-rank test. Statistical significance was defined as a *p*-value < 0.05.

## Results

### Study Population

Among a total of 92 TAK patients followed up at our center, 23 patients were recruited, mostly women (*n* = 21, 91%). At baseline, mean age was 43.8 ± 14.4 years and mean disease duration was 95.5 ± 61.3 months. Twenty-one patients (91%) were already on treatment with IFX-O and were therefore switched to CT-P13, whereas two patients (9%) were IFX-*naïve*. For both IFX-*naïve* patients, IFX-B was the first bDMARD introduced. In the *switch* group, mean duration of IFX-O therapy was 51.5 ± 37.9 months. Four patients (17%) had been previously treated with other biologics drugs, specifically tocilizumab (*n* = 3), and adalimumab (*n* = 1). At baseline, 21 patients (91%) were on glucocorticoids (mean dose, 4.8 ± 2.0 mg daily of prednisone equivalent), whereas 19 patients (83%) were on concomitant csDMARD. csDMARD dose was kept unchanged throughout the study. Mean IFX-B starting dose was 6.2 ± 1.8 mg/kg, and mean time interval was 5.8 ± 0.6 weeks. See Table 1 for more details.

**Table 1:** Baseline features of Takayasu patients enrolled in the study.

	**Total (*n* = 23)**
Female sex, *n* (%)	21 (91)
Age at diagnosis, years, mean ± SD	32.7 ± 13.3
Age at IFX-B start, years, mean ± SD	43.8 ± 14.4
Disease duration, months, mean ± SD	95.5 ± 61.3
Numano's classification, *n* (%)	
Class I	2 (9)
Class IIa	5 (21)
Class IIb	8 (35)
Class III	2 (9)
Class IV	2 (9)
Class V	4 (17)
IFX-naïve patients, *n* (%)	2 (9)
Previous therapy with other bDMARDs, *n* (%)	4 (17)
Concomitant PDN, *n* (%)	21 (91)
Concomitant PDN daily dose, mg, mean ± SD	4.8 ± 2.0
Concomitant csDMARD, *n* (%)	19 (82)
Azathioprine	4 (17)
Mycophenolate mofetil	1 (4)
Methotrexate	13 (57)
Sirolimus	1 (4)

*bDMARD, biologic disease-modifying antirheumatic drug; csDMARD, conventional synthetic disease-modifying antirheumatic drug; IFX-B, infliximab biosimilar; SD, standard deviation; PDN, prednisone*.

### Disease Activity at Baseline

**Figure 2:**
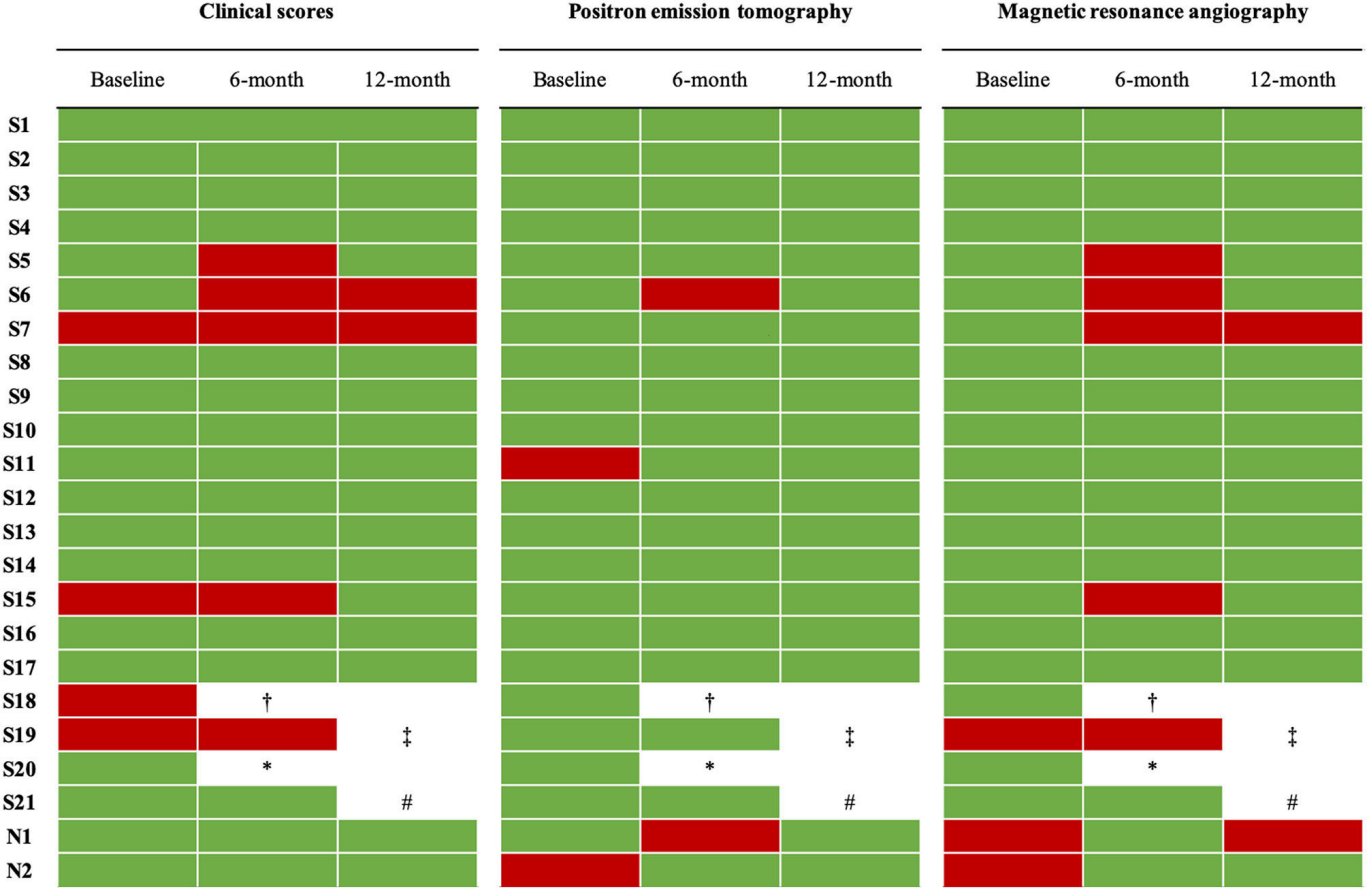
Disease activity of enrolled patients according to clinical scores and imaging parameters at baseline and at 6-month and 12-month evaluations. Clinical scores were ITAS-2020, ITAS-CRP, ITAS-ESR. According to clinical scores, Takayasu arteritis was defined active if at least one of the following conditions was present: ITAS2020 ≥ 2, ITAS-ESR ≥ 3, or ITAS-CRP ≥ 3. Green, disease not active. Red, disease active. ^†^switched to a different bDMARD at month 2 due to a clinical relapse while on the highest allowed dose of infliximab. ^‡^switched to a different bDMARD at month 6 due to persistence of disease activity while on the highest allowed dose of infliximab. *voluntarily dropped out of the study and lost to follow-up at month 3. #excluded from the study after she expressed a desire for pregnancy.

At baseline, mean ESR was 20 ± 10 mm/1h; 13 patients (56%) had a value above the normal range (i.e., > 20 mm/1 h). Mean CRP was 3.8 ± 3.1 mg/L; 9 patients (39%) had a value above the normal range (i.e., 5 mg/L). Four patients, all in the *switch* group (17%), were classified as active according to ITAS2010, ITAS-ESR, or ITAS-CRP scores. At MRA, both *naïve* patients and one *switch* patient (13%) were classified as active. At [^18^F]-FDG-PET, one *naïve* patient (50%) and one *switch* patient (9%) were classified as active. The total number of active patients according to both imaging and clinical evaluations was 7 (30%), including 2 *naïve* and 5 *switch* patients (Figure 2). Mean HAQ was 3.5 ± 5.3.

### Six-Month Evaluation

At 6 months since IFX-B introduction, study population included 21 patients. One patient voluntarily dropped out of the study at month 3 and was lost on follow-up [S20]; one patient was switched to a different bDMARD at month 2 because of an aggressive clinical relapse while on the highest allowed IFX-B dose (10 mg/kg every 5 weeks) [S18]. Notably, this patient had an active disease also when on IFX-O. Retention rate of CT-P13 at 6 months was 90.9%.

In 5 of the 21 remaining patients (24%), IFX-B dose was increased as due to inadequate disease control (patients were classified “active”); consequently, mean IFX-B dose at 6 months was 7.4 ± 2.2 mg/kg. Time interval between IFX-B infusions was kept unchanged in all patients. Among the 21 patients included in the 6-month evaluation, 19 (90.5%) were on glucocorticoids, with a significantly lower mean daily dose of prednisone equivalent compared to baseline (4.2 ± 1.9 mg at 6 months vs. 4.8 ± 2.1 mg at baseline, *p* = 0.009). Mean CRP and ESR levels did not change significantly, being 24 ± 18 mm/1 h and 8 ± 15.3 mg/L, respectively.

The number of patients classified as clinically active at 6 months according to ITAS2020 did not change (*n* = 4), whereas the number of patients classified as active according to ITAS-ESR and ITAS-CRP increased by one (*n* = 5). No *naïve* patient was active at month 6 according to clinical scores.

[^18^F]-FDG-PET showed presence of new vascular lesions in two patients (10%), and improvement or stability of pre-existing lesions in 1 (5%) and 18 (85%) patients, respectively. According to the MRA evaluation, pre-existing vascular lesions remained stable in 11 patients (50%), progressed in 5 patients (25%), and improved in 5 patients (25%). One *naïve* patient was active at [^18^F]-FDG-PET evaluation; no *naïve* patient was active at MRA evaluation.

Globally, 7 patients (1 *naïv*e and 6 *switch*) were classified as active, including the patient started on a different bDMARD at month 2 (patient S18) (Figure 2). As one of these active patients was on the highest allowed dose [S19], as per study protocol she was started on a different bDMARD. The percentage of active patients at 6 months was not significantly different from baseline (32 vs. 30%).

No patient experienced side effects during IFX-B infusion. One patient experienced grade 1 adverse event (diarrhea) and 12 patients (65%) experienced grade 2 adverse events related to the ongoing therapy. Specifically, upper airway infection (*n* = 7), herpes simplex reactivation (*n* = 4), vaginal candidiasis (*n* = 1). These events required neither hospitalization nor a modification of IFX-B therapy.

Mean HAQ score was 3.28 ± 6.42 and it was not significantly different from baseline (*p* > 0.05 for comparison in the whole group and in *naïve* and *switch* patients).

### Twelve-Month Evaluation

At 12 months since IFX-B introduction, study population included 19 patients, as one patient expressed the desire to become pregnant and was therefore excluded from the study as per protocol [S21]. Retention rate of CT-P13 at 12 months was 90.4%. In all remaining patients, both IFX-B doses and time intervals were kept unchanged compared to 6-month evaluation. Among the 19 patients included in the 12-month evaluation, 15 (79%) were on glucocorticoids, with a significantly lower mean daily dose of prednisone equivalent compared to baseline (3.3 ± 2.6 at 12 months vs. 4.8 ± 2.2 at baseline, *p* = 0.034).

Mean ESR and CRP levels were 22 ± 14 mm/1 h and 4.1 ± 5.4 mg/L, respectively, and were not significantly different from both baseline and 6-month evaluation.

Two *switch* patients had an active disease according to both ITAS-2010 and ITAS-CRP, and one of them also according to ITAS-ESR.

[^18^F]-FDG-PET showed neither new vascular uptake nor worsening of previously detected vascular uptake in all patients. MRA disclosed disease stability in 9 (47%), worsening in 2 (11%), and improvement in 8 (42%) patients. One *naïve* patient was active at MRA assessment; no *naïve* patient was active at [^18^F]-FDG-PET assessment.

Globally, 5 patients were classified as active. Two of them were the patients started on a different bDMARD at month 2 and 6. The other three patients were judged to be active according to clinical scores (*n* =1), MRA assessment (*n* = 1), or both (*n* = 1). In these 3 patients, IFX-B dose was further increased at month 12 as they were not already on the highest tolerated IFX-B dose.

The percentage of active patients at 12 months was not significantly different compared to baseline (24 vs. 30%). No patient experienced side effects during IFX-B infusion. One patient experienced grade 1 adverse event (diarrhea) and 9 patients (47%) experienced grade 2 adverse events. Specifically, herpes simplex reactivation (*n* = 5), urinary tract infection (*n* = 3), herpes zoster reactivation (*n* = 1), bacterial pneumonia (*n* = 1). These events did not require hospitalization. IFX-B infusion was delayed by 2 weeks in the patient suffering from pneumonia.

Mean HAQ score was 3.31 ± 6.35 and it was not significantly different compared to baseline (*p* > 0.05 for comparison in the whole group and in *naïve* and *switch* patients).

## Discussion

In this open-label prospective study, we investigated the efficacy and safety of CT-P13, a biosimilar of IFX, in the treatment of patients with refractory TAK. We included TAK patients already on IFX-O (defined as *switch* patients), and patients naïve to IFX-O.

In our study we observed that the percentage of active patients throughout the study period did not significantly change, as it went from 30% at baseline to 32% at 6 months and to 24% at 12 months. Notably, 12-month retention rate of CT-P13 was 90.4%, a result in keeping with what we had already previously observed in our cohort of TAK patients treated with IFX-O (78%) (10). Moreover, it is important to notice that the 2 *switch* patients started on a different bDMARD throughout the study period were both active at baseline, thus suggesting a poor efficacy of the IFX mechanism of action rather than a loss of efficacy of CT-P13 compared to IFX-O. The safety profile of CT-P13 was excellent, as no patient experienced infusion reaction and all drug-related adverse events were graded 1 or 2, with no need of CT-P13 suspension due to safety issues.

Most importantly, although the percentage of active patients did not change, a significant reduction in the mean daily dose of glucocorticoids was observed. The reduction was already evident at 6 months and was maintained at 12 months, supporting the role of CT-P13 as an effective steroid-sparing agent in refractory TAK patients.

From the point of view of imaging evaluations, in this study we decided to include both MRA and [^18^F]-FDG-PET assessments to monitor the evolution of vascular inflammatory lesions, since these two techniques can provide different and complementary pieces of information (16). The response according to [^18^F]-FDG-PET scan evaluation was satisfactory, as only two patients had active disease at 6 months, and no patient at 12 months. Moreover, it is important to notice that the two patients with an active baseline PET had an optimal response for the whole study period. In addition, in one of them daily glucocorticoid dose was reduced suggesting a positive effect of CT-P13 on [^18^F]-FDG vascular uptake reduction.

Conversely, MRA data were more controversial. Indeed, although the majority of patients showed an improvement or stability at 12 months, in 2 cases (one *switch* and one *naïve* patient) a progression of pre-existing vascular lesions was observed. Of note, among the 3 patients with an active MRA at baseline, only the 2 *naïve* patients underwent the 12-month MRA evaluation, as the *switch* patient was started on a different bDMARD.

The use of CT-P13 for the treatment of TAK is not a novelty. In 2018, Park and colleagues had already found that treatment with CT-P13 was associated with clinical, radiographic, and serological improvement in 11 TAK patients (13). However, the aforementioned study included only IFX-naïve patients, leaving open questions on the feasibility of switching IFX-O-treated TAK patients. Moreover, due to the small number of patients included, the role of CT-P13 as an effective steroid-sparing agent could not be evaluated. In our study, the majority of enrolled patients were switched from IFX-O and this allowed us to investigate whether the introduction of CT-P13 could be associated with a loss of efficacy. This point is of fundamental importance, especially considering that IFX therapy in TAK patients is usually maintained chronically due to the high risk of relapse upon discontinuation (4). A less expensive but equally effective alternative implies significant economic savings. Indeed, in a recent study including patients with inflammatory bowel disease, it was estimated that switching from IFX-O to IFX-B produced a saving of $6837 per patient-year of therapy (17).

Our study has some limitations. First, the small number of patients included prevented us from drawing clear conclusions on the efficacy of CT-P13 in refractory TAK patients. Second, due to the absence of a control group, an adequate comparison between CT-P13 and IFX-O was not feasible. Third, given the variability of patients in terms of previous therapies, we could not establish factors associated with response to CT-P13. Fourth, the limited observational period of the study did not allow to identify whether the retention rate and the immunogenicity of CT-P13 are different to IFX-O in a longer follow-up period. Nonetheless, given the rarity of the disease and the absence of prospective randomized-controlled trials on IFX in TAK patients, this study provides further and stronger evidences on the role of this drug for the management of refractory TAK patients and on the feasibility of switching from IFX-O to IFX-B.

In conclusion, in this prospective open-label trial, CT-P13 was found to have satisfying efficacy and safety profile. CT-P13 was able to control both clinical manifestations and vascular inflammation in patients with refractory TAK naïve to IFX or switched from IFX-O. No significant differences in terms of disease control were observed at 6 and 12 months after switching to CT-P13. In addition, the use of CT-P13 was not associated with an increased risk of infusion-related or generally drug-related side effects.

## Data Availability Statement

The original contributions presented in the study are included in the article/supplementary material, further inquiries can be directed to the corresponding author/s.

## Ethics Statement

This study was reviewed and approved by San Raffaele Hospital Ethics Committee. The patients/participants provided their written informed consent to participate in this study.

## Author Contributions

CC, AT, EB, GC, and LD designed the study. CC, AT, SS, and CS obtained and analyzed the data. EB took care of patients. MarP and FF performed and analyzed PET studies. MauP and FD performed and analyzed MRA studies. CC and AT drafted the manuscript. All authors contributed to the manuscript revision and approved the final version.

## Conflict of Interest

The authors declare that the research was conducted in the absence of any commercial or financial relationships that could be construed as a potential conflict of interest.

## Publisher's Note

All claims expressed in this article are solely those of the authors and do not necessarily represent those of their affiliated organizations, or those of the publisher, the editors and the reviewers. Any product that may be evaluated in this article, or claim that may be made by its manufacturer, is not guaranteed or endorsed by the publisher.
